# Imitating the cost of males: A hypothesis for coexistence of all‐female sperm‐dependent species and their sexual host

**DOI:** 10.1002/ece3.3681

**Published:** 2017-11-26

**Authors:** Christelle Leung, Bernard Angers

**Affiliations:** ^1^ Department of Biological Sciences Université de Montréal Montreal QC Canada

**Keywords:** asexual population, demographic cost, gynogenesis, paternal leakage, polyploidy, sperm dependence

## Abstract

All‐female sperm‐dependent species are particular asexual organisms that must coexist with a closely related sexual host for reproduction. However, demographic advantages of asexual over sexual species that have to produce male individuals could lead both to extinction. The unresolved question of their coexistence still challenges and fascinates evolutionary biologists. As an alternative hypothesis, we propose those asexual organisms are afflicted by a demographic cost analogous to the production of males to prevent exclusion of the host. Previously proposed hypotheses stated that asexual individuals relied on a lower fecundity than sexual females to cope with demographic advantage. In contrast, we propose that both sexual and asexual species display the same number of offspring, but half of asexual individuals imitate the cost of sex by occupying ecological niches but producing no offspring. Simulations of population growth in closed systems under different demographic scenarios revealed that only the presence of nonreproductive individuals in asexual females can result in long‐term coexistence. This hypothesis is supported by the fact that half of the females in some sperm‐dependent organisms did not reproduce clonally.

## 1 INTRODUCTION

In vertebrates, most of the all‐female organisms reproduce clonally by gynogenesis, also known as pseudogamy or sperm‐dependent parthenogenesis. This asexual mode of reproduction required the sperm of a closely related species to trigger the development of unreduced eggs (Beukeboom & Vrijenhoek, 1998; Hubbs & Hubbs, 1932). In contrast with parthenogenetic organisms that do not require males, the presence of males is a *sine qua non* condition for the maintenance of all‐female sperm‐dependent species. The persistence of gynogenetic systems is a puzzling phenomenon given the twofold cost of males (Maynard Smith, 1978; Williams, 1975). For a given fecundity rate, all‐female organisms have a demographic advantage over sexual species that must invest half of their progeny in males. This may result in a rapid extinction of the sexual host and, concurrently, to the all‐female species’ own demise in absence of available sperm donors.

This paradoxical coexistence is still an intriguing phenomenon for biologists, as evidenced by the large number of ecological hypotheses proposed. These include males’ discrimination against asexual females to ensure the reproductive success of sexual females (Moore & McKay, 1971; Schlupp, 2009, 2010), lower competitive ability of asexual forms compared to their sexual counterparts to prevent proliferation of asexual individuals (Schley, Doncaster, & Sluckin, 2004) or the use of distinct ecological niches by gynogens and their sexual host (Schley et al., 2004; Schlosser, Doeringsfeld, Elder, & Arzayus, 1998; Schlupp, 2005). These hypotheses have been supported by empirical observations and, to some extent, likely play a role in the coexistence of gynogens and their sexual host. However, these hypotheses are still debated in the literature. For instance, male preferences were too weak or not detected in some host species (e.g., Barron, Lawson, & Jensen, 2016). In addition, male behavior could not fully prevent extinction, as mating with heterospecific females may increase their attractiveness (Heubel, Rankin, & Kokko, 2009; Schlupp, Marler, & Ryan, 1994). The hybrid origin of gynogens coupled to clonality may also result in a perpetual F1 heterosis and higher performances than their sexual parents (Mee, Chan, & Taylor, 2013; Scharnweber, Plath, & Tobler, 2011; Schlupp, 2005; Vrijenhoek, 1998). Finally, while niche differentiation was demonstrated in some gynogenetic systems (e.g., Greenwald, Denton, & Gibbs, 2016; Schlosser et al., 1998), niche overlap among the two reproductive forms was also observed (e.g., Heubel, 2004; Scharnweber, Plath, Winemiller, & Tobler, 2011; Scharnweber, Plath, Tobler, et al., 2011). Moreover, both sexual and asexual females will always compete for the same limited resource—the males (Hubbs & Hubbs, 1932; Schlupp et al., 1998). Lack of convincing evidence in some systems led to the development of theoretical works based on the hypothesis that if coexistence is not possible at a local scale, it can occur in a metapopulational structure (Kokko, Heubel, & Rankin, 2008), although no empirical evidence for this hypothesis has been reported yet.

Most of the previous hypotheses involved ecological interactions between sexual and asexual individuals. Rather, we propose to investigate intrinsic factors affecting the demography of asexual organisms as an alternative process that can result in coexistence in the absence of mate choice, differences in competitive ability or specific niches. Our hypothesis is that all‐female sperm‐dependent organisms have to cope with the same demographic handicap as their sexual counterparts to maintain long‐term coexistence with the related sexual host and that half of asexual sperm‐dependent offspring should not demographically contribute to the next generation, as would be the case if they were males. Such demographic handicap can result from differential fecundity rates between sexual and asexual organisms, as proposed in studies comparing the life history of both reproductive forms (Congdon, Vitt, & Hadley, 1978; Jokela, Lively, Dybdahl, & Fox, 1997; Kearney & Shine, 2005; Lamb & Willey, 1979). However, previous studies have shown that both sexual and asexual females could produce the same number of offspring (e.g., Barron et al., 2016; Schlupp, Taebel‐Hellwig, & Tobler, 2010; Weeks, 1995). We therefore introduce the hypothesis that both sexual and asexual females display the same fecundity rate, but half of asexual offspring do not provide gynogenetic progeny to the next generation, for instance by being sterile. We used a simulation‐based study to assess the establishment and persistence of a gynogenetic system under these different demographic scenarios.

## 2 METHODS

Based on the Wright–Fisher demographic model, simulations of population growth were performed to assess the establishment of an asexual sperm‐dependent population within a sexual population and their persistence through time.

For all simulations, there was no sexual preference and sperm availability was unlimited as long as at least one sexual male was present. To reflect natural conditions, variance was allowed, and the number of individuals was rounded down to integers. At the beginning of each simulation, populations started with a number of individuals equal to the carrying capacity, set at *N*_t_ = 1,000. To simulate the formation of a gynogenetic lineage, the simulations began with a single asexual individual (*Na* = 1) while the population was dominated by sexual individuals (*Ns* = 999).

There were discrete generations. At each generation, the number of offspring produced by each female followed a Gaussian distribution with mean μ  =  20 and variance σ^2^ = 2. For sexual populations, the proportion of females was set at 50% with variance σ^2^ = 0.01, while three scenarios were simulated for asexual populations: (i) no handicap: both sexual and asexual females displayed the same fecundity rate; (ii) a fecundity handicap: asexual females displayed a fecundity rate twice lower than sexual females; and (iii) a sterility handicap: both sexual and asexual females displayed the same fecundity rate, but half of asexual offspring were sterile with variance σ^2^ = 0.01. Individuals survived proportionally to the number of offspring produced. However, this proportion was subject to random fluctuation: the number of sexual or asexual individuals in the next generation was given by the binomial distribution B(*N*_t_, *P*), where the number of trial was the carrying capacity (*N*_t_) and the probability of success the proportion of sexual or asexual individuals (*P*). Random sampling continued at each generation until either sexual or asexual form was lost (*P *=* *0). Simulations ran for 10,000 generations and 100 replicates were performed. For each scenario, the number of populations that persisted until 10,000 generations was counted.

Furthermore, different variances around 50% of asexual sterility were set (from σ^2^ = 0.01 [*SD* = 10%] to σ^2^ = 0.05 [*SD* = 22%]) to determine how much noise can be tolerated while maintaining a long‐term coexistence with the sexual host. We also carried out these different scenarios by changing the proportion of sterile asexual individuals to μ  =  40% and μ  =  60% and with different relative proportions of asexual individuals (ranging from 5% to 95%) at the beginning of each simulation.

## 3 RESULTS

**Figure 1:**
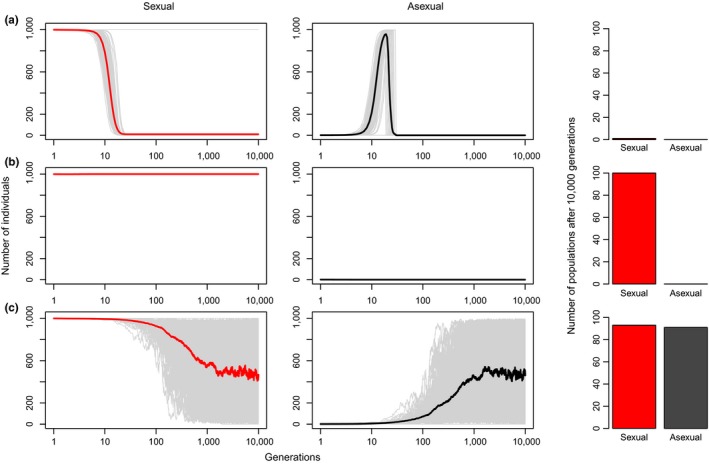
Simulations of population's growth in coexisting sexual and asexual organisms under three different scenarios. All populations started with *Ns* = 999 sexual individuals and *Na* = 1 asexual individual, and only the reproductive conditions of asexual females varied among scenarios: (a) no handicap: both sexual and asexual females displayed the same fecundity rate; (b) fecundity handicap: asexual females displayed a fecundity rate twice lower than sexual females; (c) sterility handicap: both sexual and asexual females displayed the same fecundity rate, but half of asexual offspring were sterile. Gray lines represent the dynamism of each simulated population; red and black lines represent the mean of sexual and asexual individuals, respectively, at each generation

As expected, a total absence of demographic handicap on asexual reproductive form resulted in its exponential growth and invasion to the detriment of the sexual form, even if simulations began with only a single asexual individual (Figure 1a). Such scenario is therefore not a sustainable biological system because it led to the extinction of all sexual populations (and the elimination of males) in less than 20 generations. Similarly, when asexual females displayed a fecundity rate lower than that of sexual females, a high proportion of sexual populations survived, but no asexual populations were able to persist (Figure 1b). A lower fecundity alone is thus not sufficient to explain the establishment and stable coexistence of gynogenetic systems, as producing an equal number of females by both forms is only beneficial for sexual organisms.

**Figure 2:**
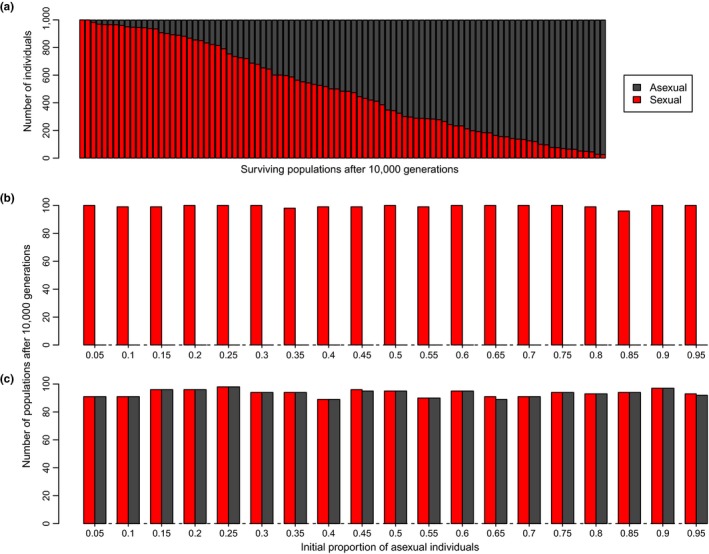
Effect of different initial proportion of asexual individuals on the persistence of sexual and asexual populations. (a) Relative proportion of asexual individuals after 10,000 generations under the sterility handicap scenario. Number of surviving populations after another 10,000 generations as a function of different initial proportion of asexual individuals under the (b) fertility and (c) sterility handicap scenarios

In a sterility handicap scenario, asexual females displayed the same fecundity rate as sexual females, but half of asexual offspring were sterile. This situation resulted in the persistence of both sexual and asexual populations in more than 90% of the replicates (Figure 1c). The relative proportion of sexual versus asexual individuals in populations that persisted after 10,000 generations was extremely variable (Figure 2a). However, the initial proportion of asexual individuals did not influence the result of the scenario simulated. Under the fertility handicap scenario, only sexual populations persisted after 10,000 generations (Figure 2b). In contrast, the same number of sexual and asexual populations persisted for another 10,000 generations when both reproductive forms were afflicted by the same demographic cost, regardless of the initial relative proportion of asexual individuals (Figure 2c).

Increasing the variance around 50% of asexual sterility up to σ^2^ = 0.05 did not modify the number of persisting populations, either for asexual or sexual individuals. This result indicates that the proportion of sterile individuals can vary between 28% and 72% (50% ± [*SD* = 22%]) without effecting the long‐term persistence.

Alternatively, changing the proportion of sterile asexual individuals below or above 50% always resulted in the decay of asexual populations. A mean below μ  =  50% sterile asexual individuals led to the extinction of sexual populations (similar to Figure 1a), while only sexual populations survived with more than μ  =  50% sterile asexual individuals (similar to Figure 1b), in accordance with the violation of Fisher's principle for sex ratio (Fisher, 1930).

## 4 DISCUSSION

Simulations of population growth under different demographic scenarios revealed that the presence of nonreproductive individuals is a relevant hypothesis, at least theoretically, to explain a long‐term coexistence of sexual and asexual organisms.

The handicap induced by 50% sterile individuals in gynogens has two consequences that jointly mediate the stable coexistence of asexual females with their sexual counterparts. First, producing asexual individuals that do not leave offspring, as do sexual males, reduces the twofold demographic advantage of gynogens. However, it is important to note that only reducing the fitness of asexual individuals is not sufficient for long‐term coexistence, according to the second condition: the occupation of niches by individuals, even sterile, limits the proliferation of sexual individuals. The finite size of the population determined by the carrying capacity resulted in a random variation of the relative abundance of both sexual and asexual forms throughout generations, especially when the population size is small (Charlesworth, 2009). Therefore, the presence of sterile asexual individuals was essential to limit the number of sexual individuals within the population. Indeed, if individuals were not viable (fecundity handicap) instead of being sterile, their absence would favor the sexual form because of their higher proportion during reproduction.

Interestingly, the coexistence of both forms may occur regardless of the initial proportions of sexual and asexual individuals, from a single individual (such as when a new gynogenetic lineage occurred from hybridization) to a massive invasion of a lineage (such as following the colonization of a site). Our model is then flexible enough to account for the heterogeneity in the relative proportion of asexual sperm‐dependent individuals and their sexual host observed in natural populations (Bogart & Klemens, 2008; Bohlen & Ráb, 2001; Choleva, Apostolou, Ráb, & Janko, 2008; Heubel, 2004; Schlosser et al., 1998; Vergilino, Leung, & Angers, 2016).

The literature presents some support for the result that half of the gynogen's progeny do not reproduce clonally. The clonality in a gynogenetic system is related to the absence of fusion between male and unreduced female pronuclei. However, fertilization (paternal leakage) has been reported in numerous gynogenetic systems and results in triploid individuals (Alves, Coelho, & Collares‐Pereira, 2001; Bogart & Lichts, 1986; Choleva et al., 2012; Goddard, Dawley, & Dowling, 1989; Itono et al., 2007; Mérette, Bourret, & Turgeon, 2009; Nanda et al., 1995; Schultz & Kallman, 1968). Triploidy is usually associated with sterility in most vertebrates (Benfey, 1999). Sterility resulting from paternal leakage was observed in laboratory experiments involving the Amazon molly *Poecilia formosa* (Lampert et al., 2007; Nanda et al., 1995). Similar experiments were performed on the spined loach hybrid *Cobitis elongatoides‐taenia* and resulted in one F1 hybrid female that produced a mixed clutch of di‐ and triploid offspring at a rate of 50% (Choleva et al., 2012). In *Chrosomus eos‐neogaeus* gynogens, paternal leakage occurs with a rate of 50% (Goddard & Dawley, 1990), and triploid individuals do not reproduce clonally (Goddard & Schultz, 1993). Furthermore, the rate of triploid hybrid *C. eos‐neogaeus* in natural populations did not significantly differ from 50%: 120 diploids of 366 hybrids from 41 lineages and 56 sites (χ^2^ = 3.085; 1 *df*;* p* = .079; Vergilino et al., 2016) and 52 diploids of 119 hybrids from a single lineage (χ^2^ = 1.891; 1 *df*;* p *= .169; Massicotte, Whitelaw, & Angers, 2011). Interestingly, the high number of triploid individuals observed in those two punctual, independent samplings (67% and 56%, respectively) remained in the range of variation tolerated for a long‐term coexistence with their sexual host. Indeed, while a mean of 50% of asexual individual sterility is required over all generations, high variance around this 50% is also possible for the coexistence of both reproductive forms, as included in the simulations.

The demographic handicap hypothesis allows further examination of the oddities observed in some gynogenetic systems. For instance, we can revisit the questions of why half of the gynogens’ progeny in *C. eos‐neogaeus* are triploid individuals that do not reproduce clonally (or at all) (Goddard & Schultz, 1993). Such waste of individuals could not be explained by previous hypotheses (but see Beukeboom & Vrijenhoek, 1998 for a possible role of triploid individuals), but this question finds a satisfactory explanation in our model as these extras are essential to the persistence of the lineage by preventing sexual individuals from occupying the niche of the asexual forms.

In other systems, the situation in natural populations is often more complex, and numerous additional biotypes can coexist. For instance, gynogenetic triploid lineages have been observed in *Poecilia formosa* (Schories, Lampert, Lamatsch, De León, & Schartl, 2007; Turner, Balsano, Monaco, & Rasch, 1983) and *Cobitis elongatoides‐taenia* (Choleva et al., 2012; Janko et al., 2007) in addition to diploid gynogenetic lineages. Because such triploid individuals reproduce asexually, they should also cope with the same demographic handicap as diploids to ensure the coexistence with their sexual host. Interestingly, paternal leakage is also possible for triploid gynogens, resulting in tetraploid individuals (Lampert, Lamatsch, Fischer, & Schartl, 2008), but the rate of this paternal genome inclusion and its consequences on individual fertility remains to be assessed. However, such gynogenetic triploids do not occur frequently in nature (Lampert, Lamatsch, Epplen, & Schartl, 2005; Schories et al., 2007), and all attempts to synthesize fertile triploid lineages as those found in some natural populations have resulted in sterile triploid individuals (Choleva et al., 2012; Lampert et al., 2007). These studies suggest that the majority of females resulting from hybridization events produced eggs that are inclined to paternal leakage, but at different probabilities. The persisting gynogenetic systems might therefore be the result of selection that only favored lineages for which paternal leakage occurs at 50% to ensure the sterility of half of the asexual individuals.

Interestingly, the sterility handicap hypothesis and the ecological hypotheses proposed to explain the long‐term coexistence between all‐female sperm‐dependent organisms and their sexual hosts are not mutually exclusive. For instance, in the case that gene expression is additive, triploid individuals are expected to be phenotypically intermediate to the sexual species and diploid gynogens. The elevation of ploidy following paternal leakage has thus been proposed as a means to tighten up the differences between sexual females and gynogens (Beukeboom & Vrijenhoek, 1998). Therefore, in addition to preventing the proliferation of the sexual form, triploid individuals could also favor gynogens’ reproduction when males display a preference for conspecific females. As another example, triploid females in *C. eos‐neogaeus* complex can occasionally give birth to cytoplasmic hybrid individuals that are composed of a nuclear genome of *C. eos* but a mitochondrial DNA of *C. neogaeus* (i.e., cybrids; Angers & Schlosser, 2007; Goddard & Schultz, 1993). In such a case, triploid females do not reproduce clonally and, as a consequence, do not contribute to increasing the demography of the asexual population. Interestingly, those cybrids can reproduce sexually and serve as sexual host for gynogens. However, the rate and the ecological conditions for cybrid formation remain to be assessed. Indeed, according to Barron et al.'s (2016) theoretical model, the systematic production of cybrids by triploid individuals, even if the proportion of triploid is as less as 5%, would result in the total replacement of the ancestral sexual *C. eos* populations. However, previous studies reported the presence of *C. eos* in sympatry with gynogenetic and cybrid individuals in various natural populations throughout the *C. eos‐neogaeus* distribution (Angers & Schlosser, 2007; Mee & Taylor, 2012; Vergilino et al., 2016). This suggests that the occurrence of cybrids from triploid individuals is rather an infrequent process.

While the lack of published data hamper formally testing our hypothesis in the several known gynogenetic systems, the scarce results concerning the rate of paternal leakage as well as the fate of these individuals are consistent with our predictions that half of the progeny of gynogens cannot reproduce clonally, while the exact mechanisms responsible for sterility remain to be elucidated. The demographic cost associated with the production of males, therefore, appears to not be restricted to gonochoric organisms but could extend to some asexual organisms derived from sexual species. Furthermore, the key role of sterile individuals for the persistence of closely related fertile individuals represents an evolutionary role analogous to sterile workers in social insects (Hamilton, 1972) and sterile flowers in some plants (Jin et al., 2010). In conclusion, the hypothesis of imitating the cost of males by gynogens presented in this study may provide fuel to further investigate the exciting but unresolved question of coexistence between all‐female sperm‐dependent and sexual species.

## CONFLICT OF INTEREST

None declared.

## AUTHOR CONTRIBUTIONS

CL and BA designed research; CL performed research and analyzed data; CL and BA wrote the paper.
